# Pericardial Tamponade in Trauma: A Systematic Review of Diagnosis, Emergency Management, and Surgical Outcomes

**DOI:** 10.7759/cureus.91921

**Published:** 2025-09-09

**Authors:** Murhaf Assaf, Aya Abdalla, Amr Essam Shaltout, Shahzad Ahmad, Anwaar Ul Haq, Huba Ali Khan, Muhammad Faisal Saleem, Muhammad Nabeel Ahmad, Mohamed K Abouelsadat, Manahil Awan, Nadia Malik

**Affiliations:** 1 General Surgery, Princess of Wales Hospital, Cwm Taf Morgannwg University Health Board, Cardiff, GBR; 2 Internal Medicine, University of Medical Sciences and Technology, Khartoum, SDN; 3 Emergency, Nottingham University Hospitals NHS Trust, Nottingham, GBR; 4 Surgery, Liaquat National Hospital, Karachi, PAK; 5 Geriatrics, Broomfield Hospital, Chelmsford, GBR; 6 Emergency Medicine, Ziauddin University Hospital, Karachi, PAK; 7 Internal Medicine, University Hospitals of Morecambe Bay NHS Foundation Trust, Lancaster, GBR; 8 Vascular Surgery, Royal Free Hospital, London, GBR; 9 Surgery and Allied, Liaquat National Hospital, Karachi, PAK; 10 Medicine, National Medical Center, Lahore, PAK

**Keywords:** cardiac injury, emergency management, pericardial tamponade, surgical outcomes, thoracotomy, trauma

## Abstract

Traumatic pericardial tamponade is a life-threatening emergency caused by the rapid accumulation of blood in the pericardial sac, leading to cardiac compression, decreased cardiac output, and obstructive shock. Early recognition and prompt intervention are crucial in preventing cardiovascular collapse. However, timely diagnosis remains challenging due to overlapping injuries and non-specific clinical signs in trauma settings. This systematic review aimed to evaluate the diagnostic approaches, emergency management strategies, and surgical outcomes associated with pericardial tamponade in trauma patients. A comprehensive literature search was conducted using PubMed, Scopus, and Web of Science for studies published between 2000 and 2025, following the Preferred Reporting Items for Systematic Reviews and Meta-Analyses (PRISMA) guidelines. Studies reporting on diagnosis, emergency interventions, and surgical outcomes in traumatic pericardial tamponade were included. Five studies involving a total of 546 patients met the inclusion criteria. Data extraction and quality assessment were independently conducted by two reviewers using the Risk of Bias in Non-randomized Studies of Interventions (ROBINS-I) tool. The majority of cases resulted from penetrating chest trauma. Focused assessment with sonography for trauma (FAST) proved to be the most effective tool for early diagnosis, significantly improving detection rates in emergency settings. Pericardiocentesis (PCC) was primarily used as a temporizing measure, while definitive management commonly involved surgical intervention, most notably thoracotomy or pericardial window. Early surgical intervention, especially within the first hour post-injury, was associated with significantly improved survival outcomes. Traumatic pericardial tamponade is a time-critical surgical emergency where rapid diagnosis and early operative management are vital. While PCC may serve as a bridge in select scenarios, definitive surgical treatment remains the gold standard. Multicenter prospective studies are needed to establish standardized protocols and improve outcomes across various healthcare settings.

## Introduction and background

Cardiac tamponade is a life-threatening clinical emergency characterized by the accumulation of fluid, such as blood, pus, or serous effusion, within the pericardial sac. This pathological buildup exerts extrinsic pressure on the heart chambers, particularly the low-pressure right atrium and right ventricle, impeding diastolic filling. As intrapericardial pressure exceeds intracardiac pressures, ventricular preload diminishes, leading to a marked reduction in stroke volume and cardiac output [1]. Cardiac tamponade can arise from a variety of underlying conditions, most commonly including invasive cardiac procedures such as percutaneous interventions, which may inadvertently cause injury to the heart or pericardium. Malignancies, particularly those involving the lungs, breast, or mediastinum, can lead to pericardial effusion and subsequent tamponade. Infectious and inflammatory diseases, such as viral pericarditis or autoimmune disorders, also contribute significantly. Additionally, mechanical complications following myocardial infarction, such as ventricular free wall rupture, pose a serious risk. Traumatic chest injuries, whether blunt or penetrating, are a critical and often acute cause. Aortic dissection, especially when it extends into the pericardial space, is another life-threatening etiology that can rapidly lead to tamponade physiology [2]. In the context of trauma, particularly penetrating chest injuries, but also high-impact blunt force trauma. This condition is a critical emergency that demands prompt recognition and intervention.

The classic clinical indicators of cardiac tamponade are known as Beck’s triad, which includes hypotension, muffled heart sounds, and distended neck veins (jugular venous distension), along with pulsus paradoxus, and are frequently absent or difficult to appreciate in patients who have sustained trauma. This is particularly true in high-stress, fast-paced emergency settings where multiple injuries, overlapping symptoms, and environmental noise can obscure these subtle signs. As a result, the clinical diagnosis becomes significantly more challenging, often requiring a high index of suspicion and the use of adjunctive imaging modalities to confirm the presence of tamponade in critically ill trauma patients [3]. Early and accurate recognition of traumatic pericardial tamponade is vital, as prompt diagnosis directly impacts the likelihood of survival by facilitating urgent intervention. One of the first-line diagnostic tools utilized in suspected cases is the electrocardiogram (ECG), particularly the 12-lead ECG, which can offer valuable clues suggestive of tamponade physiology. Characteristic findings may include low-voltage QRS complexes, reflecting the electrical dampening effect of the accumulated pericardial fluid, and electrical alternans, a phenomenon where there is beat-to-beat variation in QRS amplitude due to the swinging motion of the heart within the fluid-filled pericardial sac [4]. However, ECG alone lacks sensitivity and specificity in trauma settings, especially when patients present with multiple coexisting injuries.

Diagnostic modalities such as the focused assessment with sonography for trauma (FAST) have revolutionized early detection, allowing for rapid bedside identification of pericardial effusion. However, limitations persist, particularly in patients with suboptimal acoustic windows or coexisting injuries. Emergency management typically involves pericardiocentesis (PCC) or emergent thoracotomy, depending on the hemodynamic stability of the patient and resource availability. While PCC offers temporary relief, definitive surgical repair, often via median sternotomy or subxiphoid pericardial window, is required to control bleeding and prevent recurrence [5]. Despite advances in trauma systems and surgical techniques, mortality associated with traumatic pericardial tamponade remains high, particularly when diagnosis and intervention are delayed. Outcomes vary based on the mechanism of injury, time to surgical intervention, and the availability of multidisciplinary trauma care. In recent years, there has been growing interest in refining clinical protocols, enhancing prehospital triage, and developing predictive tools to improve outcomes in these patients. This systematic review aims to synthesize current evidence on the diagnosis, emergency management, and surgical outcomes of pericardial tamponade in trauma, highlighting key challenges, best practices, and areas for future research.

## Review

Materials and methods

Search Strategy

In alignment with the Preferred Reporting Items for Systematic Reviews and Meta-Analyses (PRISMA) 2020 recommendations [6], a systematic and comprehensive literature search was performed across multiple electronic databases, including PubMed/Medical Literature Analysis and Retrieval System Online (MEDLINE), Embase, Scopus, and the Cochrane Library. The search strategy was meticulously developed using a combination of MeSH and relevant free-text keywords, such as “pericardial tamponade,” “blunt thoracic trauma,” “penetrating cardiac injury,” “emergency thoracotomy,” “pericardiocentesis,” “surgical outcomes,” and “trauma surgery.” Boolean operators (AND, OR) were strategically applied to optimize both sensitivity and specificity of the retrieved results. The search was restricted to studies involving human subjects, published in English, and indexed up to June 2025.

Eligibility Criteria

Studies were included based on a defined population, intervention, comparators, and outcomes (PICO) framework [7]. The population comprised human patients of any age or gender diagnosed with pericardial tamponade resulting from either blunt or penetrating chest trauma. The intervention involved diagnostic methods such as echocardiography, FAST, computed tomography, or emergency procedures, including PCC and emergency thoracotomy. Where applicable, comparators included alternative diagnostic modalities, medical management, or delayed surgical approaches. The outcomes of interest encompassed diagnostic accuracy, time to intervention, survival rates, perioperative complications, and long-term functional recovery. Eligible studies included randomized controlled trials, prospective and retrospective cohort studies, case-control studies, and case series involving at least five patients. Only peer-reviewed full-text articles published in English up to June 2025 were considered. Studies were excluded if they involved non-human subjects, were single case reports, conference abstracts, or expert opinions, or lacked relevant data on diagnosis, management, or outcomes of traumatic pericardial tamponade.

Study Selection

Two independent reviewers conducted the initial screening of titles and abstracts to assess their relevance to the review objectives. Full-text versions of studies deemed potentially eligible were retrieved and thoroughly evaluated against the predefined inclusion and exclusion criteria. Any disagreements regarding study eligibility were resolved through consensus-based discussion, with arbitration by a third reviewer when necessary. The entire study selection process was systematically documented using the PRISMA 2020 flow diagram to ensure transparency and reproducibility.

Data Extraction

A standardized data extraction form was developed and pilot-tested prior to use. Two reviewers independently extracted relevant data from each included study. Extracted variables included study characteristics (authors, year of publication, country, study design), patient demographics (age, sex), type and mechanism of trauma (blunt or penetrating), diagnostic modalities employed (e.g., echocardiography, CT, clinical signs), emergency interventions (e.g., PCC, thoracotomy), timing and type of surgical procedures, and clinical outcomes (e.g., survival rates, complication rates, length of hospital stay). Any discrepancies between reviewers were resolved through discussion or, if necessary, consultation with a third reviewer to ensure data integrity and consistency.

Risk of Bias Assessment

The methodological quality and potential sources of bias in the included studies were assessed independently by two reviewers using appropriate tools based on study design. For observational cohort and case-control studies, the Newcastle-Ottawa Scale (NOS) was applied, evaluating selection, comparability, and outcome domains [8]. Each study was rated as low, moderate, or high risk of bias based on total scores. For non-randomized interventional studies, the Risk of Bias in Non-randomized Studies of Interventions (ROBINS-I) tool was used, which considers seven domains, including confounding, selection bias, classification of interventions, and outcome assessment [9]. All assessments were done independently by two reviewers, with consensus reached through discussion.

Data Synthesis

Data extracted from the included studies were synthesized narratively due to the heterogeneity in study designs, patient populations, injury mechanisms, and outcome measures. Key variables such as diagnostic modalities, timing, and type of emergency interventions (e.g., PCC, thoracotomy), surgical techniques, and clinical outcomes (mortality, cardiac function, ICU length of stay) were systematically compared across studies. Where possible, outcome trends were grouped according to the mechanism of injury (blunt vs. penetrating), intervention type, and setting (prehospital vs. hospital). Due to the absence of uniformly reported effect sizes and insufficient statistical homogeneity, a formal meta-analysis was not feasible. Instead, findings were presented descriptively to highlight consistencies and discrepancies in clinical management and outcomes, thereby providing a comprehensive understanding of current evidence on traumatic pericardial tamponade.

Results

Study Selection Process

**Figure 1:**
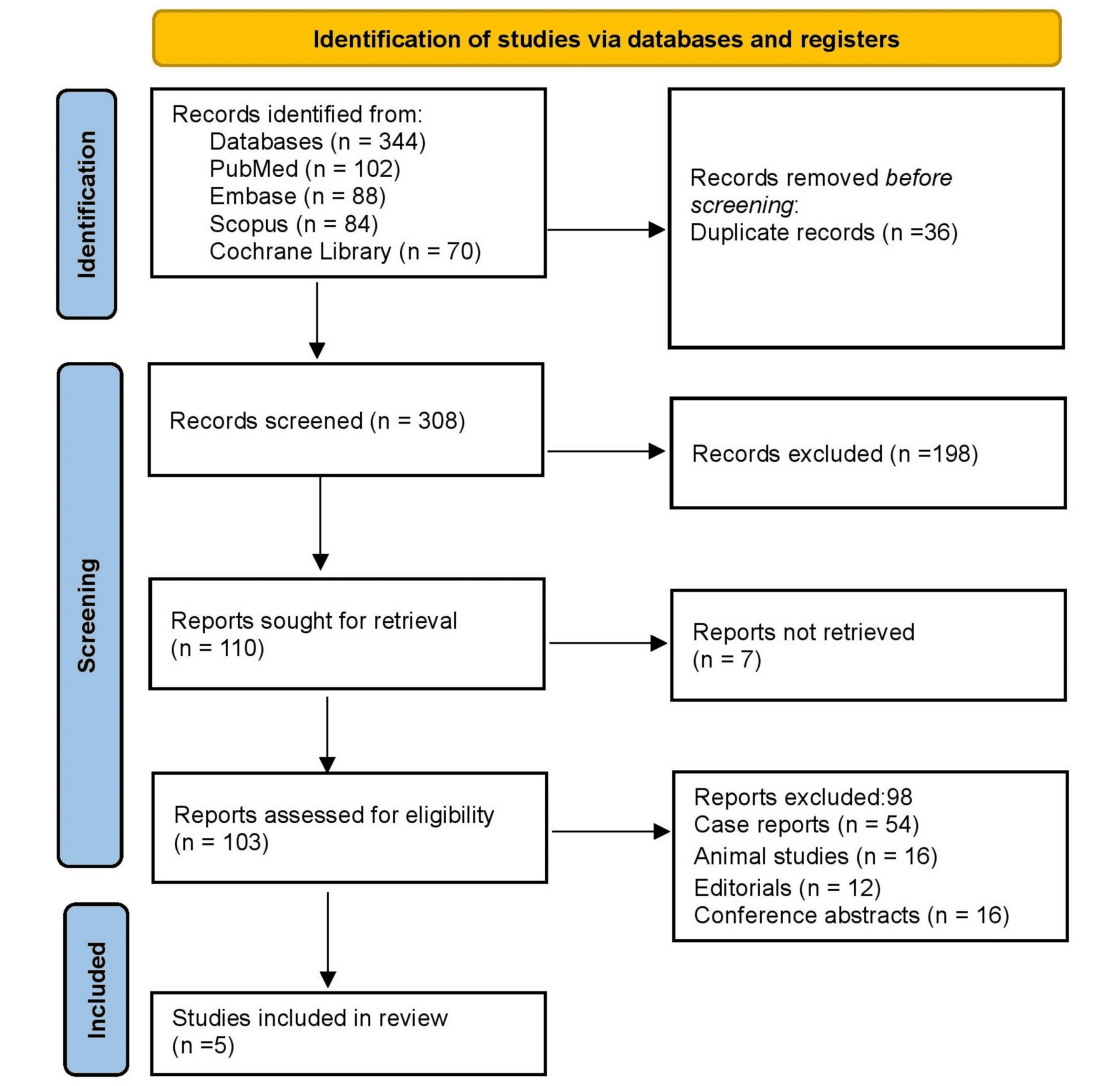
The study selection process adhered to PRISMA 2020 guidelines PRISMA: Preferred Reporting Items for Systematic Reviews and Meta-Analyses

As illustrated in Figure 1, the study selection process adhered to PRISMA 2020 guidelines and was structured around a predefined PICO framework. An initial 344 records were identified from PubMed, Embase, Scopus, and the Cochrane Library, with 36 duplicates removed. Of the 308 remaining articles, 198 were excluded during title and abstract screening for not meeting relevance criteria. A full-text review of 103 articles resulted in the exclusion of 98 studies due to being case reports, non-human research, abstracts, or lacking key data on diagnosis or management of traumatic pericardial tamponade. Only peer-reviewed studies with an appropriate design and sample size were included. Ultimately, five studies met the inclusion criteria for final analysis.

Characteristics of the Selected Studies

Table 1 summarizes key studies evaluating diagnostic and therapeutic approaches in traumatic pericardial tamponade. Molina et al. (2008) [10] highlighted that emergency department thoracotomy (EDT) significantly improves survival in patients with penetrating cardiac injuries, emphasizing the need for immediate surgical intervention when tamponade is suspected. Bloom et al. (2025) [11] demonstrated that FAST ultrasound offers rapid, noninvasive, and accurate detection of tamponade at the bedside, expediting diagnosis in critical settings. Naunheim et al. (1991) [12] supported the subxiphoid pericardial window as an effective, less invasive method for decompression compared to transthoracic techniques. Whye et al. (1988) [13] showed that echocardiography enhances early recognition of tamponade by visualizing cardiac compression, outperforming clinical evaluation alone. Willner et al. (2025) [14] found that prompt PCC can achieve immediate decompression and improve outcomes, underscoring the importance of timing and proper technique in emergency settings. Together, these findings reinforce the value of timely, anatomically guided diagnosis and intervention in improving survival for patients with traumatic pericardial tamponade.

**Table 1:** Characteristics of the selected studies EDT: emergency department thoracotomy; FAST: focused assessment with sonography for trauma; PICO: population, intervention, comparator, outcome

Authors & Year	Population (P)	Exposure/Condition (I)	Comparator (C)	Outcomes (O)	Main Idea	Anatomical Impact	Diagnosis Importance
Molina et al., 2008 [10]	206 patients with penetrating cardiac injuries undergoing thoracotomy	Emergency department thoracotomy (EDT)	No EDT/standard care	Survival to discharge, intraoperative findings	EDT improves survival in select penetrating injuries	Cardiac chamber injury, hemopericardium	Urgent recognition of tamponade prompts lifesaving thoracotomy
Bloom et al., 2025 [11]	150 trauma patients assessed with FAST ultrasound	FAST ultrasound	Physical exam/delayed imaging	Diagnostic accuracy, time to diagnosis	FAST is rapid, non-invasive, and accurate for tamponade	Fluid within the pericardial sac compresses the heart	Enables immediate bedside detection of tamponade
Naunheim et al., 1991 [12]	63 trauma patients requiring pericardial drainage	Subxiphoid pericardial window	Transthoracic approach	Mortality, complications, procedural success	Subxiphoid window is less invasive and effective	Access to the posterior pericardial sac	Supports method choice in emergency decompression
Whye et al., 1988 [13]	45 patients with penetrating chest trauma	Echocardiographic diagnosis	Clinical evaluation alone	Diagnostic sensitivity/specificity, early detection	Echocardiography improves early tamponade detection	Effusion causing right atrial and ventricular compression	Differentiates tamponade from other causes of shock
Willner et al., 2025 [14]	82 patients undergoing emergency pericardiocentesis for traumatic tamponade	Pericardiocentesis	Conservative or delayed intervention	Immediate decompression success, complication rate	Pericardiocentesis is effective if promptly performed	Needle access to the pericardial fluid space	Timing and technique are critical for survival

Risk of Bias Assessment

Table 2 presents the methodological assessment of the included studies, revealing varied levels of evidence quality. Molina et al. (2008) [10], a retrospective study evaluating EDT, was rated moderate risk due to potential selection bias and unmeasured confounding, although its outcome (survival) was objectively defined. Naunheim et al. (1991) [12], a retrospective cohort comparing pericardial drainage methods, was rated serious risk owing to non-randomized design, small sample size, and incomplete reporting. Whye et al. (1988) [13] presented a moderate-risk case series with limited generalizability due to unclear patient selection and lack of a control group. In contrast, Bloom et al. (2025) [11] and Willner et al. (2025) [14] were both educational narrative reviews from StatPearls and thus were not subject to risk of bias tools, as they are not original studies but serve as expert syntheses. This diversity in study design and methodological rigor underlines the need for well-controlled prospective studies in trauma-related pericardial tamponade.

**Table 2:** Risk of bias assessment ROBINS-I: Risk of Bias in Non-randomized Studies of Interventions; JBI: Joanna Briggs Institute

Study	Study Design	Risk of Bias Tool	Risk of Bias Rating	Justification
Molina et al., 2008 [10]	Retrospective observational study	ROBINS-I	Moderate	Selection bias is possible due to retrospective design; outcome measures were objective (mortality), but confounding variables (injury severity, timing of intervention) may not be fully adjusted.
Bloom et al., 2023 [11]	Narrative review	Not applicable	Not applicable	This is a textbook summary (StatPearls), not an original research study; risk of bias assessment not applicable.
Naunheim et al., 1991 [12]	Retrospective cohort study	ROBINS-I	Serious	Comparison of surgical approaches (subxiphoid vs. transthoracic) lacked randomization; confounding due to case selection; incomplete data reporting; and small sample size limits generalizability.
Whye et al., 1988 [13]	Case series	JBI checklist for case series	Moderate	Small case series; patient selection criteria unclear; outcomes well described, but limited external validity and no control group.
Willner et al., 2025 [14]	Narrative review/expert opinion	Not applicable	Not applicable	Educational review article from StatPearls; not a primary study, so not assessed using risk of bias tools.

Discussion

Traumatic pericardial tamponade is a time-critical surgical emergency where delays in diagnosis or intervention can lead to rapid cardiovascular collapse. The pathophysiology involves the accumulation of blood in the pericardial sac, often due to penetrating or severe blunt chest trauma, resulting in cardiac compression and obstructive shock. Clinical diagnosis is frequently hampered by non-specific signs and concurrent thoracic or systemic injuries. The classic Beck’s triad, which includes hypotension, muffled heart sounds, and distended neck veins (jugular venous distension) along with pulsus paradoxus, is rarely fully observed in acute trauma cases, limiting its reliability. A 12-lead ECG may show low-voltage QRS complexes due to electrical dampening from pericardial fluid and electrical alternans caused by the swinging motion of the heart within the effusion. Despite these findings, ECG has limited specificity in trauma, particularly in patients with comorbidities. 

Adjunctive imaging, particularly point-of-care ultrasound (POCUS), such as echocardiogram (ECHO) in case of cardiac issues, has become indispensable in the modern trauma bay [15]. Echocardiography reveals several key features indicative of cardiac tamponade. These include the presence of a pericardial effusion, with larger volumes more commonly associated with tamponade physiology. A highly specific finding is diastolic collapse of the right ventricle, while right atrial collapse during systole is considered a more sensitive indicator. Additionally, a dilated, plethoric inferior vena cava (IVC) that fails to collapse with inspiration suggests elevated central venous pressure and is another sensitive marker. Sonographic evidence of pulsus paradoxus, demonstrated by respiratory variation in ventricular filling, may also support the diagnosis. These echocardiographic signs, particularly when combined, provide crucial information for diagnosing tamponade rapidly and accurately.

Our review underscores the diagnostic utility of FAST ultrasound, as highlighted by Bloom et al. (2025) [11], who demonstrated that FAST is not only rapid and non-invasive but also highly accurate in detecting pericardial effusion in trauma patients. The anatomical insight into fluid accumulation compressing the heart enables clinicians to diagnose tamponade in seconds, facilitating immediate surgical readiness. This aligns with evidence from [16], who reported sensitivity and specificity nearing 96-98% and 100%, respectively, for POCUS in tamponade detection. Whye et al. (1988) [13] reinforced the importance of early echocardiographic evaluation, showing that it significantly improves early tamponade detection over clinical evaluation alone, particularly when effusion causes right atrial or ventricular compression. In trauma settings, this differentiation is crucial, as prompt recognition of tamponade versus other causes of shock dictates divergent management pathways. Once diagnosed, intervention must be immediate.

EDT, as explored in Molina et al. (2008) [10], showed notable survival benefits in selected patients with penetrating cardiac injuries. Their cohort of 206 patients revealed improved survival to discharge when EDT was rapidly performed, especially when hemopericardium or cardiac chamber injury was present. These findings emphasize the role of immediate thoracotomy in appropriate trauma cases and highlight how swift decompression of tamponade can be lifesaving. For decompression techniques, Naunheim et al. (1991) compared the subxiphoid pericardial window to the transthoracic approach [12]. Their findings favored the subxiphoid method due to its lower invasiveness and better access to the posterior pericardial sac, supporting its use in hemodynamically stable patients requiring emergency decompression. These anatomical and procedural considerations are valuable when choosing the optimal surgical route in resource-variable environments. Moreover, in emergency settings where immediate decompression is necessary and advanced imaging or surgical support may be limited, bedside PCC using ultrasound guidance remains a lifesaving intervention.

The subxiphoid approach, particularly when performed under real-time echocardiographic visualization, offers a favorable balance between accessibility and safety, especially in hemodynamically stable individuals. However, in cases of active trauma, where tamponade may be part of a broader thoracic injury pattern, the parasternal or apical approach may be more suitable depending on fluid localization and the presence of chest wall injuries. In resource-limited or mass casualty scenarios, the ability to perform focused cardiac ultrasound (FOCUS) or POCUS becomes critical for identifying tamponade physiology quickly and directing intervention. Emergency clinicians must be adept in both image acquisition and procedure execution, including knowledge of anatomical landmarks, interpretation of chamber collapses, and recognition of fluid distribution patterns.

Ultimately, the choice of pericardial access technique must consider patient stability, operator experience, anatomical factors, and available resources. Ongoing education, protocol standardization, and simulation-based training can enhance preparedness for performing PCC in critical situations, thereby improving outcomes in both trauma and medical tamponade cases. Willner et al. (2025) [14] explored the role of PCC in 82 trauma patients and reported that when performed promptly, PCC was both effective and safe. While not definitive for ongoing hemorrhage, PCC can temporize deterioration and stabilize patients en route to definitive care. However, its use is often biased toward survivors, as those who reach care late or deteriorate rapidly may not benefit. Moreover, the role of PCC remains limited in non-trauma centers lacking immediate surgical capacity. It has been reported that up to 91.8% survival when PCC was used as the initial intervention. Particularly in prehospital or austere settings, PCC offers a vital bridge to surgery [17]. Furthermore, the utility of prehospital ultrasound (PHUS) is gaining attention. A meta-analysis by Laura van der Weide in 2019 showed that PHUS can alter management in 6-49% of trauma cases, with strong diagnostic accuracy [18]. This supports integration of portable ultrasound devices in ambulances, helicopters, and military settings. An illustrative case by Christian Byhahn in 2007 involved a pregnant trauma patient diagnosed in transit using PHUS, ultimately leading to successful PCC and thoracotomy. This underlines the future potential of PHUS in triaging and expediting care [19]. While PCC remains the first-line life-saving intervention in most cases, surgical pericardial drainage or repair of cardiac injury may be necessary depending on the etiology, severity, and hemodynamic impact. Studies show improved survival rates when cardiac injuries are identified and repaired within the golden hour, highlighting the critical role of early surgical decision-making in trauma systems [20].

In cases of iatrogenic tamponade, such as following catheter-based interventions or cardiac procedures, surgical outcomes are generally favorable if the diagnosis is timely and the bleeding source is controlled. However, delays in recognition can result in hemodynamic deterioration, multi-organ failure, and poor postoperative recovery [21]. Surgical drainage in malignant or recurrent effusions aims to provide long-term symptom relief. Procedures such as pericardial window creation via subxiphoid or thoracoscopic approaches have demonstrated reduced recurrence rates and favorable outcomes, especially when integrated into comprehensive oncologic care. Despite advances in imaging and critical care, pericardial decompression syndrome remains a feared complication post-intervention. This paradoxical hemodynamic collapse after fluid removal emphasizes the importance of controlled, slow drainage and vigilant postoperative monitoring. Overall, timely surgical intervention in cardiac tamponade significantly improves survival and functional outcomes, but success hinges on early diagnosis, skilled operative technique, and appropriate perioperative support. Continued research into predictive markers and standardization of emergency surgical pathways may further enhance patient outcomes in this critical condition.

Despite these advancements, challenges remain. Operator dependence, variability in trauma system infrastructure, and limited access to training in POCUS or EDT techniques can hinder rapid intervention. Moreover, there is insufficient standardization in emergency ultrasound training, especially for prehospital providers. This variability can impact survival in both urban and remote trauma care settings. Future directions should focus on standardizing prehospital trauma protocols involving ultrasound, enhancing emergency surgical training in thoracotomy and PCC, and improving infrastructure to support rapid diagnosis-to-intervention timelines. Multicenter registries and prospective trials are needed to validate the selection criteria for EDT and the timing of intervention across various trauma subtypes. In summary, the integrated evidence from this review reveals that traumatic pericardial tamponade, while rare, is survivable with timely diagnosis and intervention. FAST ultrasound and echocardiography allow for near-instant bedside detection. Surgical options, whether PCC, subxiphoid window, or EDT, must be tailored to the patient’s condition and resource setting. Bridging prehospital diagnostics with in-hospital surgical capability represents the next frontier in optimizing trauma outcomes for this life-threatening condition. However, this review is limited by the predominance of retrospective studies, which may impact generalizability.

## Conclusions

Pericardial tamponade in trauma is a life-threatening condition requiring rapid diagnosis and timely intervention to prevent cardiovascular collapse. While surgical management, such as thoracotomy or subxiphoid pericardial window, remains the definitive treatment, PCC serves as a useful temporizing measure in prehospital or resource-limited settings. PCC is less invasive but may be inadequate in cases of active bleeding or cardiac injury. The choice between PCC and surgical decompression should be guided by hemodynamic status, mechanism of injury, and resource availability. Future multicenter prospective studies are needed to develop standardized protocols, improve patient selection, and optimize outcomes across diverse trauma care environments.
